# Effect of transcranial magnetic stimulation combined with transcutaneous auricular vagus nerve stimulation on mild cognitive impairment: a study protocol for a randomized controlled trial

**DOI:** 10.3389/fnagi.2025.1600921

**Published:** 2025-05-30

**Authors:** Jingjing Zhang, Jun Ma, Yao Rao, Jiali Wu, Hui Xu, Jiawei Ni, Zhiwei Zhao, Cong Wang, Chunlei Shan

**Affiliations:** ^1^Rehabilitation Center, Tongren Hospital, Shanghai Jiao Tong University School of Medicine, Shanghai, China; ^2^Shanghai Key Laboratory of Flexible Medical Robotics, Tongren Hospital, Institute of Medical Robotics, Shanghai Jiao Tong University, Shanghai, China; ^3^Yuanshen Rehabilitation Institute, Shanghai Jiao Tong University School of Medicine, Shanghai, China

**Keywords:** mild cognitive impairment, repetitive transcranial magnetic stimulation, transcutaneous auricular vagus nerve stimulation, event-related potential, P300

## Abstract

**Background:**

Non-invasive brain stimulation techniques have been widely used in patients with mild cognitive impairment (MCI) to accelerate the recovery of their cognitive functions. However, the clinical efficacy of single non-invasive stimulation techniques in treating MCI still requires further improvement. The combination of two non-invasive neuromodulation techniques can enhance the synergistic effects of the treatment. Repetitive transcranial magnetic stimulation (rTMS) regulates the cortical-subcortical network in a “top-down” manner, while transcutaneous auricular vagus nerve stimulation (taVNS) modulates the brainstem-limbic system-cortical pathway in a “bottom-up” fashion. We will combine rTMS and taVNS, anticipating synergistic regulation through dual pathways to achieve multi-level neural remodeling effects and improve MCI.

**Methods:**

This study will investigate the effectiveness of combined rTMS and taVNS therapy in improving the cognitive function of MCI patients. We will enroll 88 participants and randomly assign them to single-stimulation groups and combined-stimulation groups. The single-stimulation groups will be further randomized in a 1:1 ratio into a rTMS + sham taVNS stimulation group and a taVNS + sham rTMS stimulation group; the combined-stimulation groups will be randomized in a 1:1 ratio into an rTMS + taVNS group and an rTMS sham stimulation + taVNS sham stimulation group. All patients will receive treatment for 4 weeks. Assessments will be conducted before treatment (T0), 4-week treatment (T1), and 4-week post-treatment follow-up (T2). The primary outcome measure will be the Chinese version of the Montreal Cognitive Assessment Basic (MoCA-B), while secondary outcome measures will include the Rivermead Behaviour Memory Test (RBMT), the modified Barthel Index (MBI) for activities of daily living, and the latency and amplitude of event-related potential (ERP) P300.

**Discussion:**

This study is a clinical randomized controlled trial, which innovatively combines two non-invasive modulation techniques to improve cognitive function in patients with MCI. This study can validate the clinical efficacy of the combined TMS + taVNS stimulation, providing a theoretical basis for the application of this technology in clinical settings.

## Introduction

Mild cognitive impairment (MCI) is an intermediate state between normal cognitive function and dementia (Mian et al., 2024; van der Veere et al., 2024). It shows varying degrees of impairment in many cognitive areas such as memory, executive function, and language ability (Knopman and Petersen, 2014; Weston et al., 2011). These problems often lead to a decline in patients’ ability to live daily and an increase in psychological problems (Avery et al., 2023), which seriously increases the social and economic burden. Epidemiology has shown that the prevalence of MCI is higher in the elderly population and increases with age. MCI is particularly common in the community (Stephan et al., 2024). Early recognition and intervention are important for delaying the further decline of cognitive function. By using tools such as the Montreal Cognitive Assessment Scale, MCI can be more effectively detected, and personalized management strategies can be provided to patients (Mian et al., 2024). In conclusion, MCI is a complex clinical condition with diverse manifestations and hazards. An in-depth understanding of the clinical features of MCI and its potential risk factors is critical to developing effective interventions and improving patient outcomes.

As a non-invasive brain stimulation technique, transcranial magnetic stimulation (TMS) can improve cognitive function by regulating the excitability of the cerebral cortex. In recent years, TMS has made some progress in improving MCI. Studies have shown that repetitive transcranial magnetic stimulation (rTMS) can significantly improve overall cognitive function and memory in patients with MCI (Jiang et al., 2020). In addition, rTMS improves cognitive function by regulating neurotransmitter balance and promoting synaptic plasticity (Li and Xiao, 2024). Functional magnetic resonance imaging (fMRI) studies have shown that rTMS can alter the functional connectivity of brain regions associated with cognition, such as increasing the functional connectivity of the left dorsolateral prefrontal cortex (DLPFC) with other brain regions (Li et al., 2020). Due to significant variations in the stimulation parameters of rTMS (such as frequency, intensity, stimulation site, etc.) across different studies, the reproducibility of results is poor. In addition, most of the existing studies are focused on short-term interventions, lacking long-term follow-up data to evaluate the sustained effects of rTMS (Cirillo et al., 2023).

Transcutaneous auricular vagal nerve stimulation (taVNS) is an emerging non-invasive vagal nerve stimulation technique (Farmer et al., 2020). In 2000, Ventureyra first proposed the concept of stimulating the vagus nerve via auricular (ear) skin (Ventureyra, 2000). TaVNS can regulate neural activity in the brain by stimulating the auricular branch of the vagus nerve, thereby improving cognitive functions (Miyatsu et al., 2024). TaVNS can significantly enhance short-term memory and immediate recall abilities in healthy adult (Cibulcova et al., 2024). It can improve the duration of attention by modulating indirect physiological markers, such as salivary α-amylase and cortisol concentrations (Luna et al., 2025). By modulating the activity of the autonomic nervous system and central nervous system, taVNS can improve overall cognitive function (Kang et al., 2024). In summary, taVNS, as an emerging neuromodulation technique, holds significant potential in improving cognitive functions.

No previous studies have reported the combined use of rTMS and taVNS for MCI. The rationale for integrating rTMS with taVNS in this study lies in their synergistic modulation of brain networks through dual regulatory pathways to improve cognitive function. High-frequency rTMS enhances DLPFC excitability via long-term potentiation (LTP) mechanisms. As a key hub for cognitive control, DLPFC activation exerts top-down regulation on cognition-related brain regions, thereby improving cognitive performance. Concurrently, taVNS activates Aβ fibers in the auricular branch of the vagus nerve, transmitting signals to the nucleus tractus solitarius (NTS). This initiates neuroplastic effects through both the locus coeruleus-noradrenergic pathway and the limbic system. The combined use of these two modalities aims to amplify the holistic regulatory effects on cognitive networks through a dual-pathway approach, encompassing both “top-down” and “bottom-up” mechanisms.

Electroencephalography (EEG) is an important tool for the early diagnosis of MCI. It can record the brain’s electrical activity in real time, reflecting the neural synchronization and desynchronization mechanisms (McBride et al., 2014). Patients with MCI often exhibit a weakening of the alpha rhythm and an enhancement of the theta rhythm, and these changes are closely associated with cognitive decline (Babiloni et al., 2021). In event-related potentials (ERPs), specific components such as N200 and P300 exhibit delayed occurrence in patients with MCI. Furthermore, MCI patients show prolonged P300 latency and reduced amplitude, indicating a slowing of cognitive processing speed (Kamal et al., 2021). EEG and ERPs provide critical insights into the neurophysiology associated with MCI.

Therefore, we plan to conduct a prospective, randomized controlled clinical trial. We hypothesize that the combination of rTMS and taVNS may produce synergistic effects, further enhancing the improvement in cognitive function. In addition to administering cognitive behavioral assessments, we will incorporate EEG to explore the neurophysiological mechanisms of this combined therapy.

## Methods

### Study design

**FIGURE 1:**
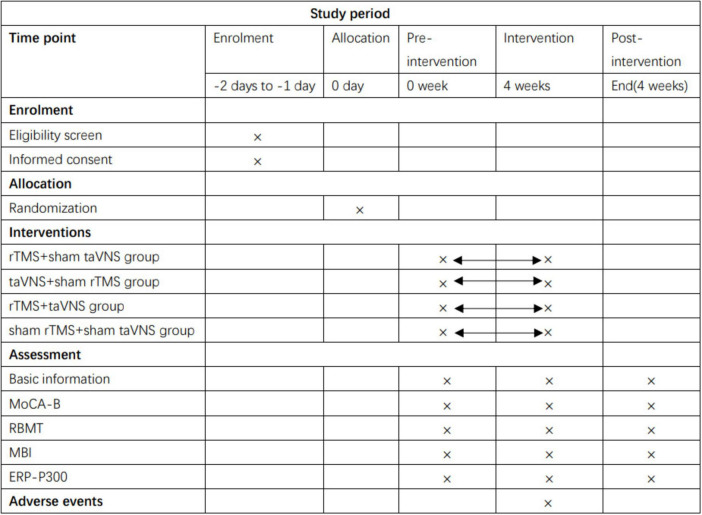
Schedule of enrollment, interventions and assessments. rTMS, repetitive transcranial magnetic stimulation; taVNS, transcutaneous auricular vagus nerve stimulation; MoCA-B, Montreal Cognitive Assessment Basic; RBMT, The Rivermead Behaviour Memory Test; MBI, modified Barthel Index; ERP-P300, Event-Related Potential P300.

**FIGURE 2:**
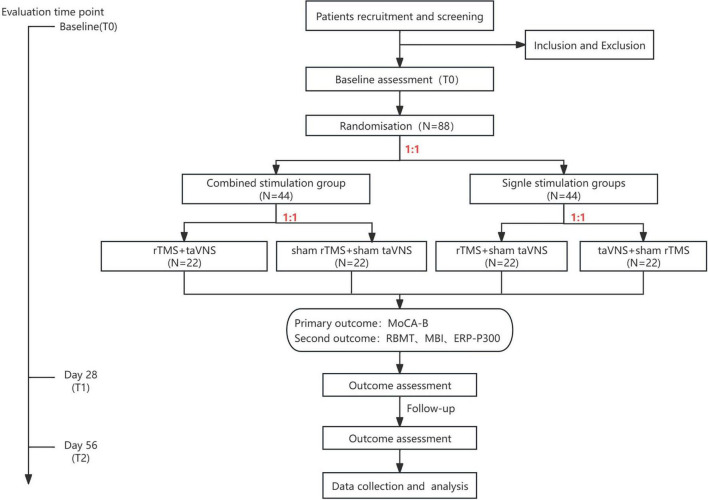
Study flow diagram. rTMS, repetitive transcranial magnetic stimulation; taVNS, transcutaneous auricular vagus nerve stimulation; MoCA-B, Montreal Cognitive Assessment Basic; RBMT, The Rivermead Behaviour Memory Test; MBI, modified Barthel Index; ERP-P300, Event-Related Potential P300.

The study is a prospective, randomized, controlled clinical trial (Figures 1, 2) that has been registered at the Chinese Clinical Trial Registry (ChiCTR2100049851).^1^

### Recruitment of participants

Participants will be recruited from the clinic, inpatient ward, or long-term care facilities near the Department of Rehabilitation, Tongren Hospital, Shanghai Jiao Tong University School of Medicine between January 2025 and December 2025. Residents who meet the inclusion criteria will be recruited.

### Diagnostic criteria

The diagnosis was based on Jak/Bondi’s criteria (Bondi et al., 2014). Participants were diagnosed as MCI if one of the following three criteria was met: (1) he or she had an impaired score, defined as > 1 standard deviation (SD) below the age-corrected normative mean, on both measures within at least one cognitive domain (i.e., memory, language, or speed/executive function); (2) he or she had one impaired score, defined as > 1 SD below the age-corrected normative mean, in each of the three cognitive domains sampled; or (3) he or she had a score on the Functional Assessment Questionnaire (FAQ) = 9 (Gloth et al., 1995) indicating dependence on three or more daily activities.

### Inclusion criteria

(1) Meet the “core” criteria for MCI, as defined by the National Institute on Aging-Alzheimer’s Association workgroups on diagnostic guidelines for Alzheimer’s disease (Albert et al., 2011);
(2) Age between 45 and 80 years;
(3) Right-handed;
(4) A Clinical Dementia Rating (CDR) score of 0.5;
(5) Voluntarily agreeing to participate in this study and signing the informed consent form.

### Exclusion criteria

(1) Cognitive dysfunction caused by other reasons (such as medications, vascular conditions, Parkinson’s disease, frontal-temporal lobe degeneration, alcohol dependence, tumors, epilepsy, and hydrocephalus);
(2) History of stroke or intracranial space-occupying lesions;
(3) Other psychiatric disorders (such as depression or severe affective disorders);
(4) Substance abuse, moderate-to-severe hypertension, or systemic diseases (such as acute renal failure or pulmonary infection);
(5) Visual or auditory impairments preventing completion of neuropsychological testing;
(6) Contraindications to rTMS treatment, such as metallic implants in the body or cardiac pacemakers;
(7) Contraindications to taVNS treatment, such as cochlear implants or other implanted electronic devices, conduction block arrhythmias, and asthma;
(8) Individuals with skin lesions or infections in the ear area.

### Randomization and allocation

A statistical professional will use MATLAB (version 2020b, MathWorks Inc., Natick, United States) to generate 88 random integers in a randomized sequence. Each patient will be assigned a unique random number based on their enrollment order. The first randomization will divide patients into a combined stimulation group (1–44) and a single stimulation group (45–88) at a 1:1 ratio. Patients in the combined stimulation group will be further randomized at a 1:1 ratio into a real stimulation subgroup (1–22) and a sham stimulation subgroup (23–44). Similarly, patients in the single stimulation group will be randomized into an rTMS + sham taVNS subgroup (45–66) and a taVNS + sham rTMS subgroup (67–88) at a 1:1 ratio. Allocation concealment will be implemented using opaque sealed envelopes. Researchers will open the envelopes sequentially according to enrollment order, and detailed records of the opener and time of unsealing will be maintained.

### Assignment of interventions: blinding

It should be noted that complete blinding of both researchers and participants to the allocation of intervention targets is not feasible due to the inherent visibility of stimulation targets. Therefore, blinding will be selectively implemented, extending exclusively to the assessors and statisticians responsible for data collection and final statistical analyses.

Unblinding of group division will occur in situations where the staff deem this necessary for participant safety.

### Interventions

All participants will receive routine cognitive training.

RTMS will be undergone using a Magventure Rapid stimulator, connected with a figure-of-eight coil (cool-B65) having a 70-mm diameter (MagPro X100, Denmark), generating 6 T as the maximum output. rTMS intensity is set up to a maximum of 90% of the resting motor threshold (RMT) for each participant. Throughout the procedure, subjects will remain in a supine position and will be instructed to remain motionless to ensure precise targeting of the stimulation site. The coil is positioned over the left DLPFC (F3 point according to the EEG 10-20 system). The daily sessions are applied with 1500 rTMS pulses at 10 Hz in short bursts of 50 pulses. In addition, the sham procedure is delivered through the same device with a specially designed coil to create identical noise as real rTMS without any electromagnetic energy. The treatment will be applied once a day for 4 weeks with rest on the weekends (4 weeks of rTMS therapy).

The taVNS device (BC 102-IV, BrainClos, Shenzhen, China) channel will be connected to two silver chloride electrodes (with an outer diameter of 7 mm). Both the anode and cathode of the taVNS will be placed inside the left cavum conchae of the ear, with the cathode positioned medially, 0.5 cm away from the anode. The skin around the left ear will be wiped with alcohol to remove any excess oil and to ensure the best possible conductivity. Stimulation parameters will be adjusted for (1) wave density: 25 Hz; (2) wave width: 500 μs; (3) Intensity: range from 0 to 6 mA in a 30-s on/30-s off cycle, adjusted to the strongest sensation that can be tolerated without pain;(4) duration: 30 min/session, daily, 5 days/week, for 4 weeks. All procedures performed in the sham taVNS group are identical to those in the taVNS group, but the instrument will administer a constant electrical current of 0.5 mA for 30 s. Subsequently, the current intensity is progressively diminished to a null level. If a participant reports no sensation, the researcher will check the device and its connection, reassure the participant of proper device connectivity, and remind them that sensation could vary depending on stimulation parameters.

Throughout the trial, participants will be requested to actively report adverse events (AEs), including but not limited to: skin irritation/erythema, dizziness, transient palpitations, or nausea potentially associated with taVNS; headache, scalp discomfort, tinnitus, or seizure-like episodes potentially associated with rTMS. Each reported AE will be documented in the participant’s case report form (CRF). In the event of a serious AE (SAE) deemed causally related to the intervention, the subject will be withdrawn from the trial and referred for appropriate medical care by the protocol-mandated SAE management algorithm.

### Demographic data collection

Demographic characteristics of all participants including age, gender, educational level, as well as medical history, medication list, and comorbidities will be documented.

### Neuropsychological assessment

A set of neuropsychological tests will be used to assess cognitive function and behavior of all the participants, and their activities of daily living will also be measured. The assessments will be performed at baseline, 4-week treatment, and 4-week post-treatment follow-up.

The Chinese version of the Montreal Cognitive Assessment Basic (MoCA-B) is a cognitive function screening tool tailored for individuals with low educational attainment, demonstrating high cultural adaptability (Chen et al., 2016; Julayanont et al., 2015). The total score is 30 points, with a score of ≥ 26 indicating normal cognitive function and a score of ≤ 26 suggesting impaired cognitive function. The cutoff scores for identifying MCI are adjusted according to the number of years of education: ≤ 19 points for those with ≤ 6 years of education, ≤ 22 points for those with 7-12 years of education, and ≤ 24 points for those with > 12 years of education.

The Rivermead Behaviour Memory Test (RBMT) is a behavioral memory assessment tool developed by the Rivermead Rehabilitation Centre in Oxford, England. It primarily evaluates patients’ memory function and consists of 12 items (Wilson et al., 1989). The total score is 24 points. The grading standards are as follows: 0–9 points indicate severe memory impairment, 10–16 points indicate moderate impairment, 17–21 points indicate mild impairment, and 22–24 points indicate normal memory function.

Modified Barthel Index (MBI) is used to assess the patient’s activities of daily living, including 10 items, including grooming, bathing, eating, toileting, and dressing (Mahoney and Barthel, 1965). The scoring standard is as follows: 100 were completely independent; 95–75 were classified as mild dysfunction; 70–50 were classified as moderate dysfunction; 50–25 were classified as severe dysfunction and obvious life dependence; ≤ 20 was totally dependent in life.

### Event-Related Potential

**FIGURE 3:**
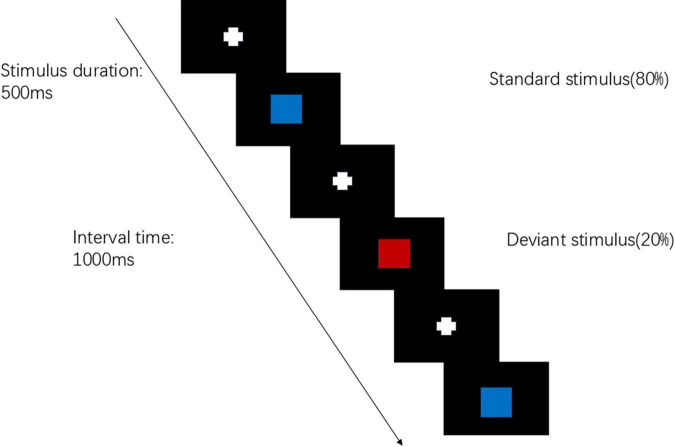
Visual P300 test behavioral experiment paradigm.

The visual P300 test will be conducted using the Chinese NianTong 32-channel ERP detection system. The test procedure employs the visual “oddball” paradigm. Stimulus materials are programmed using E-prime 3.0 software, with the deviant stimulus being a “red square” (accounting for 20%) and the standard stimulus being a “blue square” (accounting for 80%). These two different stimulus images are randomly presented on an LCD display, with a stimulus presentation time of 500 ms and an inter-stimulus interval of 1,000 ms. The target stimulus appears 40 times with a probability of 20%. A black background is used uniformly to enhance contrast and reduce visual fatigue. The subject is required to respond to the deviant stimulus each time (by clicking the left mouse button) (Figure 3). The test duration is approximately 10 min. Data acquisition is performed using eConScan_Aio software, with an AC sampling mode and a sampling frequency of 1,000 Hz to collect the raw EEG signals. The impedance between the electrodes and the subject’s skin was consistently maintained below 5 kΩ.

The analysis indicators generated after data processing include the P300 latency and amplitude. The P300 latency refers to the time interval from the onset of the stimulus signal to the peak of the waveform. The P300 amplitude is measured as the vertical distance (i.e., the peak value) from the waveform peak to the baseline, using the bilateral mastoids as the reference. The P300 component exhibits a near-central distribution on the scalp, primarily at the electrodes Fz (midline frontal site), Cz (midline central site), and Pz (midline parietal site), which are the main locations for P300 distribution. During data analysis, the P300 amplitude and latency at the Fz, Cz, and Pz electrodes are analyzed.

### Outcomes

The primary outcome is the MoCA-B score. Secondary outcomes include RBMT, MBI, P300 latency and amplitude. Any adverse events occurring during the intervention period, such as dizziness, pain, skin redness, and itchiness, will be reported by research assistants and managed by experienced physicians. We will assess the causality between adverse events and the treatment, as well as the severity of the adverse events. Serious adverse events will be reported to the ethics committee.

### Sample size calculation

The sample size was calculated using Power Analysis and Sample Size software version 15.0 (NCSS, LLC). The Factorial analysis of variance test is used for the sample size calculation of four groups. By referring to the previous literature (Wang et al., 2022). on rTMS or taVNS in the treatment of MCI, rTMS stimulation MoCA-B score is 16; taVNS stimulation MoCA-B score is 18; we estimate that the sham group MoCA-B score is 14 and the combined stimulation group MoCA-B score is 22. The target effect size is with 80% power (β = 0.20) and a type I error of 5% (α = 0.05). Considering a dropout rate of 10%, a total of 88 participants is necessary. In this study, we intend to recruit 88 participants, with 22 participants in each group.

### Quality control and quality assurance

At least three dementia specialists will work together to examine the participants and provide a diagnosis for each participant. All data will be monitored and reviewed by the principal investigator or research coordinators. Training will be provided to all researchers. Consistency coefficients in scoring assessment scales between researchers should be no less than 0.85. Data entry will be verified by a second researcher in the team. To protect participant confidentiality, only supervisors, researchers of this study, and the ethics committee will be authorized to access the personal information and medical records of the participants.

### Statistical methods

In this study, statistical analyses will be conducted using IBM SPSS Statistics 26. We will follow the intention-to-treat (ITT) principle for the primary analysis. For participants with partially missing outcome data (e.g., missed follow-up assessments), we will apply multiple imputation using chained equations under the assumption of data missing at random (MAR). Sensitivity analyses will be conducted using both complete-case analysis and per-protocol sets to assess robustness. Given the inclusion of multiple secondary outcomes (RBMT, MBI, P300 latency/amplitude), we will apply Bonferroni correction to adjust for multiple testing. Specifically, the significance threshold for secondary outcomes will be set as 0.0125. The study employs a 2 × 2 factorial design with rTMS (yes/no) and taVNS (yes/no) as independent factors. To examine the main effects and interaction effects of rTMS and taVNS on outcomes, we will use two-way repeated-measures ANOVA or mixed-effects models, depending on the distribution and structure of the data. For longitudinal outcomes measured at T0, T1, and T2, we will fit linear mixed-effects models (LMMs) with fixed effects for group, time, and their interaction, and a random intercept for participants to account for within-subject correlation. Additionally, we will explore including baseline covariates such as age, sex, and baseline cognitive score (e.g., baseline MoCA-B) in the models to improve precision and control for potential confounding. A *P* < 0.05 will be considered statistically significant.

### ERP-P300

Data processing will be conducted using MATLAB R2022b. The EEG raw data preprocessing will be performed using the EEGLAB toolbox. The preprocessing steps mainly include: localizing electrodes, removing unused electrodes, referencing, filtering, segmenting, and baseline correcting the data, as well as removing artifacts and performing independent component analysis (ICA) (Zhuang et al., 2023). After preprocessing the ERP data, the amplitude and latency of the P300 wave will be analyzed at the Fz, Cz, and Pz electrodes.

The significance level for statistical analysis will be set at 5% (*P* < 0.05). The researchers conducting the data analysis will be blinded to the allocation and interventions.

### Ethics and dissemination

This study protocol has been approved by the Ethics Review Committee of Shanghai Tongren Hospital (No. 2021-047-01). Study-related information will be provided by the Rehabilitation Department of Shanghai Tongren Hospital. The purpose, objectives, inclusion criteria, and procedures of the research will be explained in detail. The research team will offer face-to-face consultations to all potential participants and their guardians to address any questions they may have prior to signing the informed consent forms. Written informed consent will be obtained from all participants. Participants are allowed to withdraw from the study at any time, and reasons for withdrawal will be documented. The results of this study will be published in peer-reviewed journals.

## Discussion

In this study, we intend to investigate the effects of combined rTMS and taVNS on MCI patients. Our study is designed as a randomized controlled trial aiming to evaluate the effectiveness and safety of this combined therapeutic approach in improving cognitive functions.

RTMS is a non-invasive brain stimulation technique that has been extensively studied in recent years for its potential to improve cognitive function. Based on the recommendations of the International Federation of Clinical Neurophysiology (IFCN) guidelines (2014-2018) (Lefaucheur et al., 2020), we selected 10 Hz rTMS targeting the left DLPFC as the stimulation protocol for this study. The selection of parameters in this study is also supported by findings from high-quality clinical research. Research has shown that high-frequency rTMS can enhance memory function in elderly MCI patients (Drumond Marra et al., 2015). A meta-analysis study revealed that stimulating the DLPFC with 10 Hz can significantly improve cognitive function in MCI patients (Licht et al., 2023; Xie et al., 2021). RTMS promotes the recovery of cognitive function by altering cortical excitability, modulating neural plasticity, and facilitating neural network reorganization (Zhang et al., 2022).

TaVNS has been utilized to enhance cognitive function as a potential non-invasive therapeutic approach. Research indicates that taVNS can enhance cognitive function by modulating the activity of both the autonomic nervous system and the central nervous system (Kang et al., 2024). TaVNS can improve cognitive performance in patients with MCI, and such improvements can persist for a period of time after the intervention has ended (Wang et al., 2022). In addition, the effects of taVNS in improving cardiovascular function and regulating the autonomic nervous system have also been confirmed, which may further support its underlying mechanisms in enhancing cognitive function (Antonino et al., 2017). Given the current lack of standardized stimulation parameters for taVNS in improving MCI, this study referenced prior literature (Wang et al., 2022; Yao et al., 2022) to adopt parameters that balance neuromodulatory efficacy and safety. Should preliminary or formal studies reveal suboptimal parameter efficacy, we will conduct additional parameter-screening trials to compare the differential effects of various pulse width and frequency combinations. Future research must further delve into the optimization of taVNS stimulation parameters.

The P300 component of event-related potentials (ERPs) serves as a crucial indicator in cognitive neuroscience research. It is widely utilized to assess attention and cognitive processing (Asaumi et al., 2014). The P300 is typically elicited through the oddball paradigm. The latency of the P300 is related to the time taken for stimulus evaluation and response preparation. The amplitude of the P300 is associated with the amount of attentional resources invested in the task (Raggi et al., 2024). Research indicates that significant changes in the latency and amplitude of the P300 are observed in patients with MCI and Alzheimer’s Disease (AD), which are associated with pathological aging and cognitive impairment (Paitel et al., 2021). Intervening in patients with MCI through rTMS may lead to improvements in the latency and amplitude of the P300, thereby reflecting an enhancement in cognitive function (Chen et al., 2024). TaVNS failed to significantly modulate the latency and amplitude of the P300 in healthy volunteers, which may be attributed to insufficient stimulation parameters or too short a stimulation duration (Gadeyne et al., 2022). Further research is still required in the future to verify its therapeutic effects.

The innovation of this study lies in the first-time application of combining rTMS with taVNS in patients with MCI. We hypothesize that this dual intervention may synergistically amplify the therapeutic effects through both top-down and bottom-up mechanisms.

However, this study also has some limitations. Firstly, the relatively small sample size may limit the generalizability of the results. Additionally, although we employed a double-blind design, there may still be a risk of unblinding due to technical reasons. Future research should consider increasing the sample size and further exploring the impact of different stimulation parameters on the therapeutic effects.

In conclusion, this study will provide preliminary evidence for the application of combined rTMS and taVNS in patients with MCI. This will offer important theoretical foundations and practical guidance for the development of more effective treatment strategies for MCI.

## Footnotes

1
http://www.chictr.org.cn
